# Supplementary material for: Evaluation of Cell-Free Synthesized Human Channel Proteins for In Vitro Channel Research
Source: Membranes (Basel). 2022 Dec 30;13(1):48. doi: 10.3390/membranes13010048 (PMC9861611; doi:10.3390/membranes13010048)
Supplement: Supplementary file 1 [file membranes-13-00048-s001.zip › Supplementary files/Fig S2.pdf]

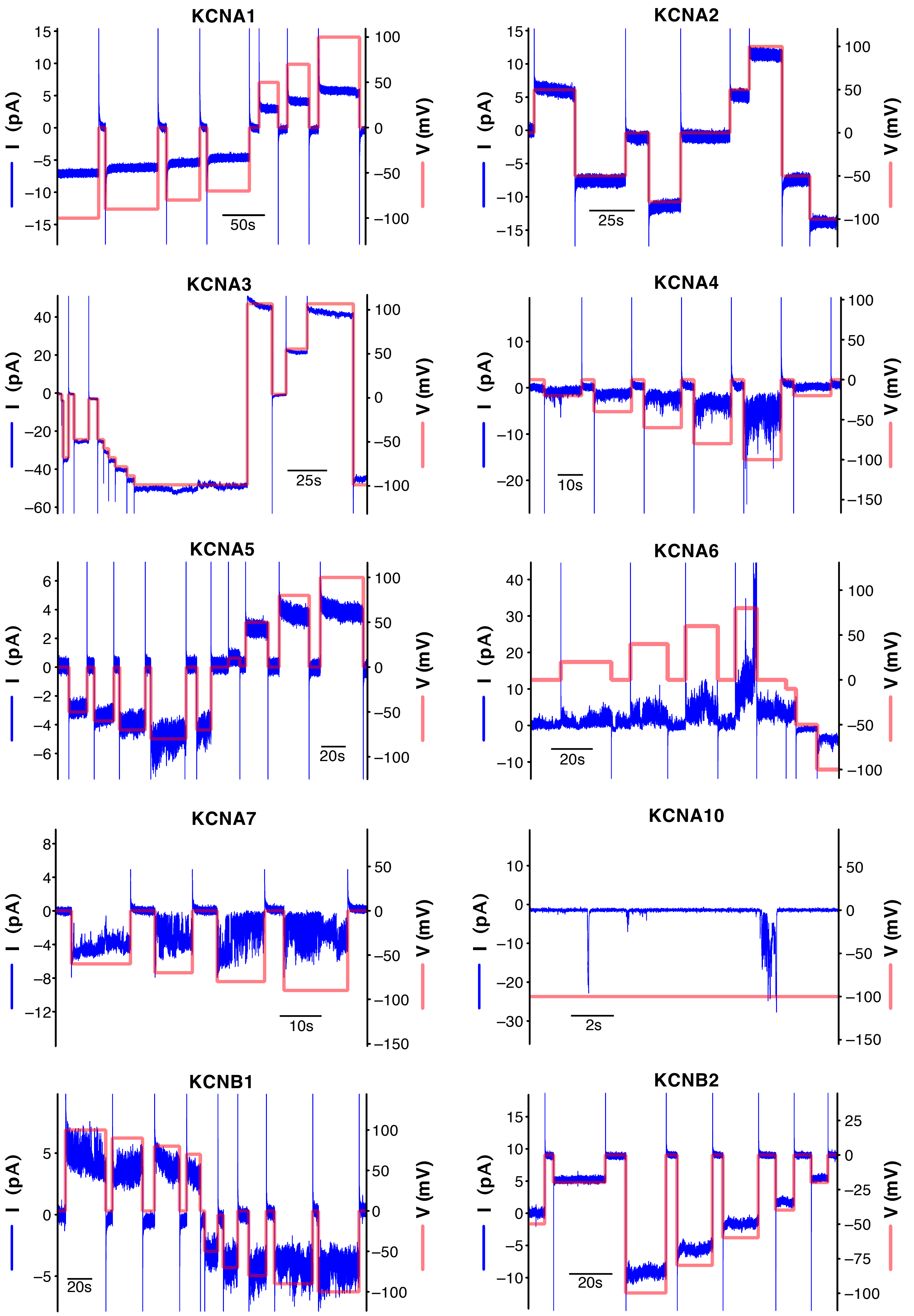

Figure S2. Representative current signals of voltage-gated potassium ion channels (1 of 4 pages)

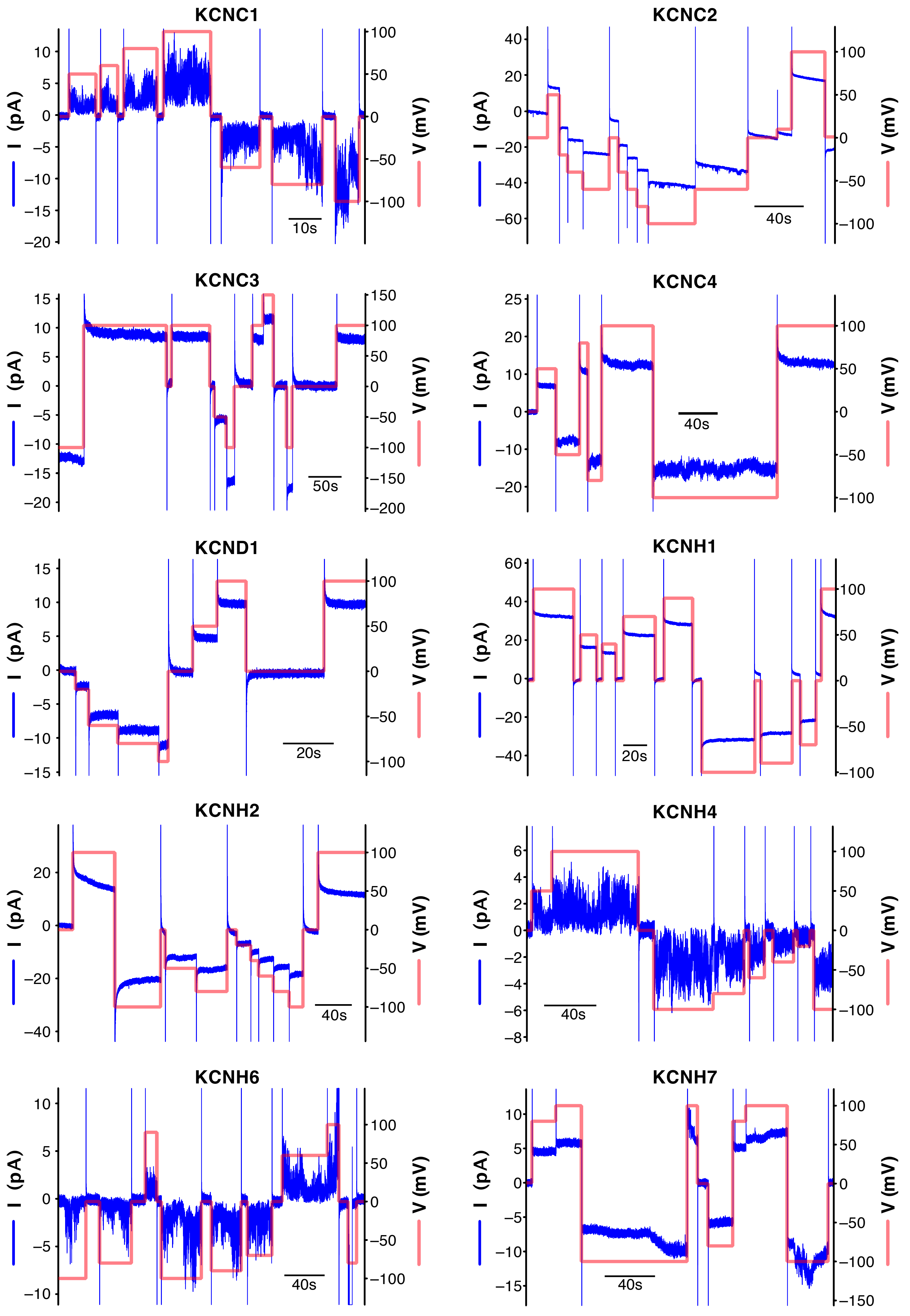

Figure S2. Continued. (2 of 4 pages)

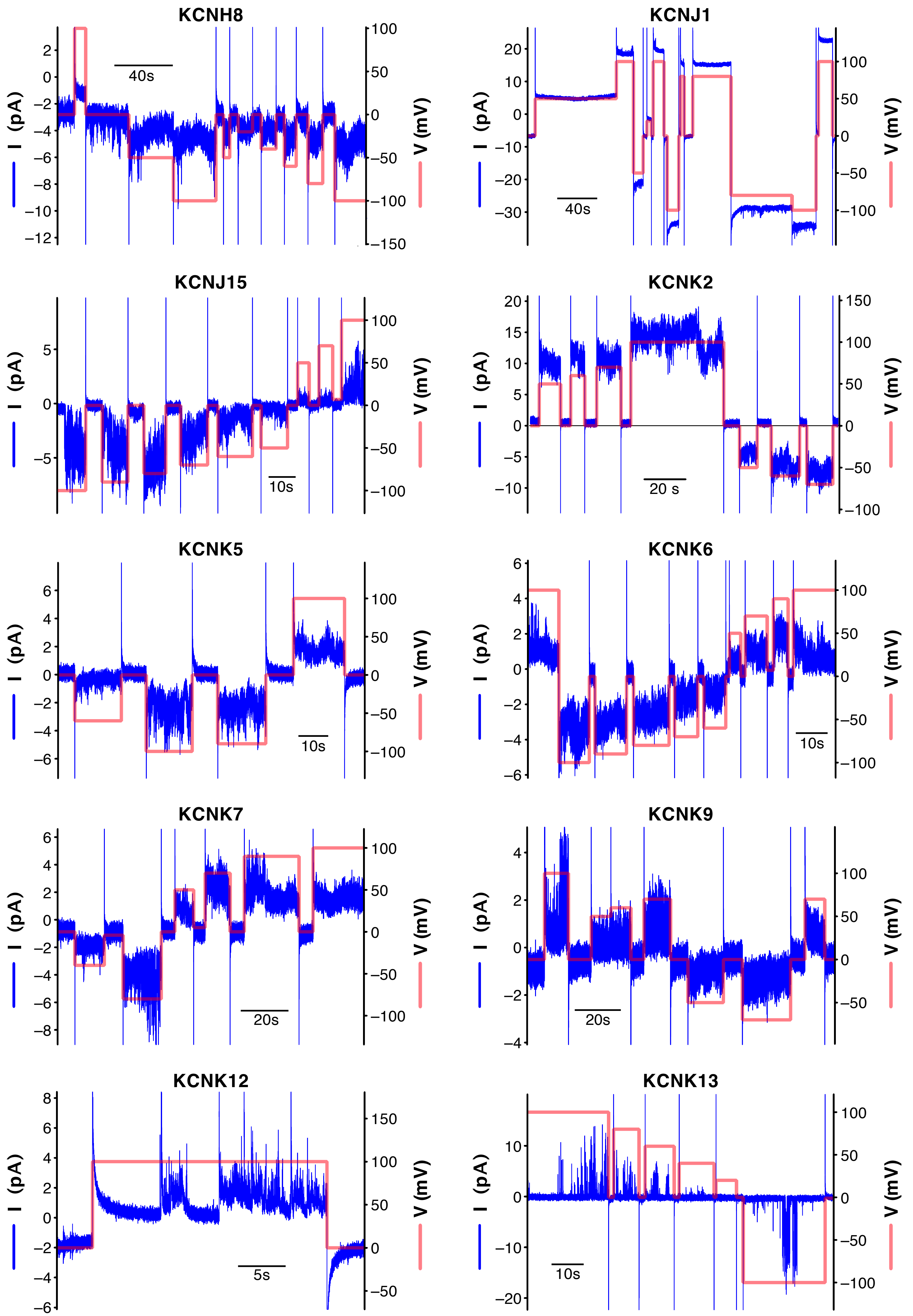

Figure S2. Continued. (3 of 4 pages)

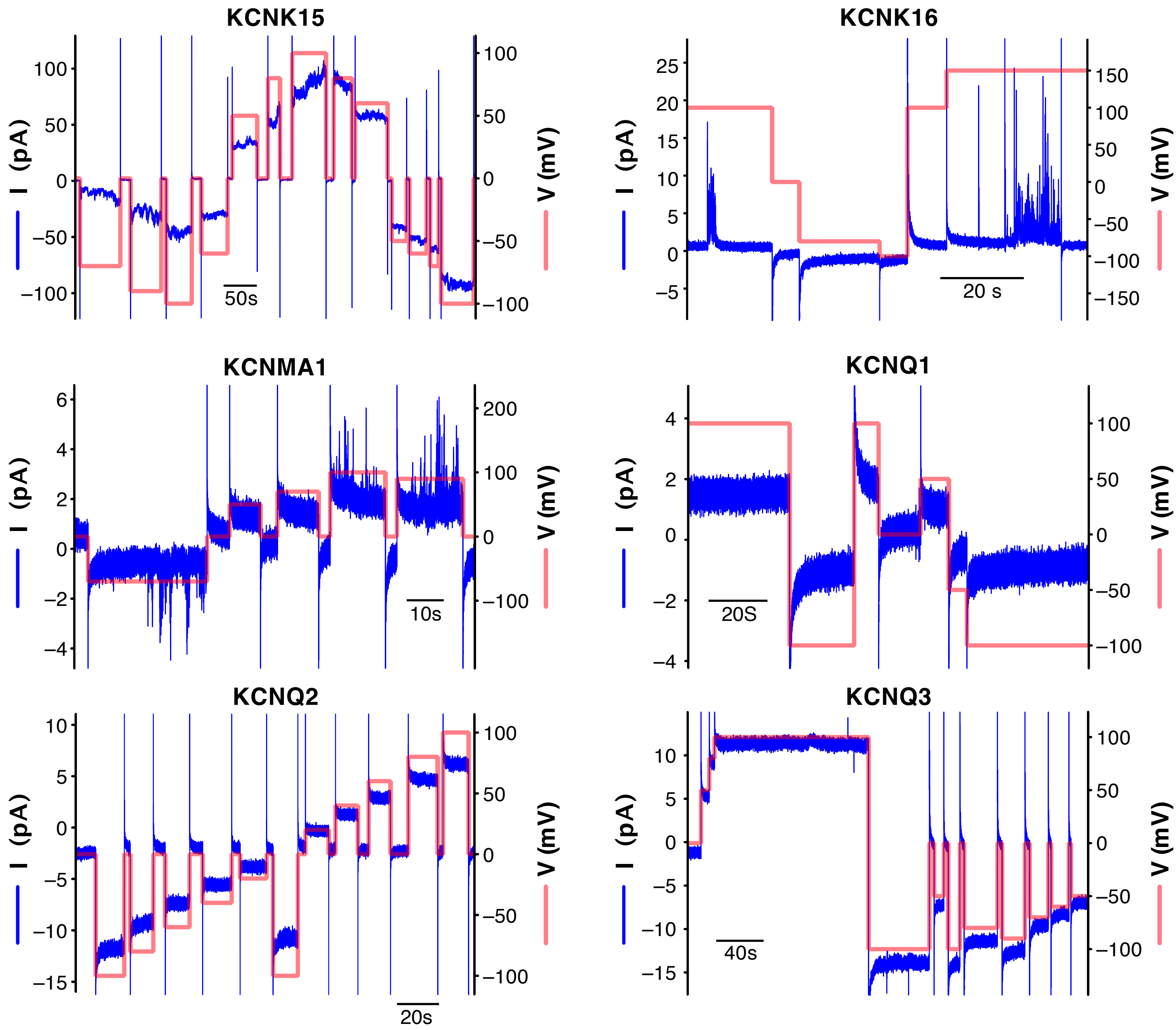

Figure S2. Continued. (4 of 4 pages)
