# Supplementary material for: Evaluation of Cell-Free Synthesized Human Channel Proteins for In Vitro Channel Research
Source: Membranes (Basel). 2022 Dec 30;13(1):48. doi: 10.3390/membranes13010048 (PMC9861611; doi:10.3390/membranes13010048)
Supplement: Supplementary file 1 [file membranes-13-00048-s001.zip › Supplementary files/Fig S1.pdf]

A

ATGGCCGAAAAAGCCCCTCCTGGCCTGAACAGAAAGACCAGCAGAAGCACCCTGAGCCTGCCTCCAGAGCCTGTGGACATCATCAGAAGCAAGACCT
GCTCTCGGCGCGTGAAGATCAATGTCGGCGGCCTGAATCACGAGGTGCTGTGGCGAACACTGGACAGACTGCCCAGAACCAGACTGGGCAAGCTGAG
AGACTGCAACACCCACGAGAGCCTGCTGGAAGTGTGCGACGACTACAACCTGAACGAGAACGAGTACTTCTTCGACAGACACCCTGGCGCCTTCACC
AGCATCCTGAACTTCTACAGAACCGGCAAGCTGCACATGATGGAAGAGATGTGCGCCCTGAGCTTCGGCCAAGAGCTGGATTACTGGGGCATCGACG
AGATCTACCTGGAAAGCTGCTGCCAGGCCAGATACCACCAAAAGAAAGAACAGATGAACGAGGAACTGCGGCGCGAGGCCGAGACTATGAGAGAAAG
AGAGGGCGAAGAGTTCGACAACACATGCTGCCCCGACAAGCGGAAGAAGCTGTGGGATCTGCTGGAAAAGCCCAACTCTAGCGTGGCCGCCAAGATC
CTGGCCATCGTGTCCATCCTGTTCATCGTGCTGAGCACAATCGCCCTGTCTCTGAACACCCTGCCTGAGCTGCAAGAGACAGACGAGTTCGGCCAGC
TGAACGACAACAGACAGCTGGCCCATGTGGAAGCCGTGTGTATCGCCTGGTTCACCATGGAATACCTGCTGCGGTTCCTGAGCAGCCCCAACAAGTG
GAAGTTCTTCAAGGGCCCACTGAACGTGATCGACCTGCTGGCCATCCTGCCTTACTACGTGACCATCTTCCTGACCGAGAGCAACAAGAGCGTGCTG
CAGTTCCAGAACGTGCGGAGAGTGGTGCAGATCTTCCGGATCATGCGGATCCTGAGAATCCTGAAGCTGGCCAGACACAGCACAGGCCTCCAGTCTC
TGGGCTTCACCCTGCGGAGAAGCTACAATGAGCTGGGCCTGCTGATCCTGTTTCTGGCCATGGGCATCATGATCTTCAGCAGCCTGGTGTTCTTCGC
CGAGAAGGACGAGGACGCCACCAAGTTCACCTCTATTCCCGCATCTTTTTGGTGGGCCACCATCACCATGACCACCGTCGGCTACGGCGACATCTAC
CCCAAAACACTGCTGGGCAAGATCGTCGGCGGACTGTGTTGTATTGCCGGCGTGCTGGTTATCGCCCTGCCTATTCCTATCATCGTGAACAACTTCA
GCGAGTTCTACAAAGAGCAGAAGCGGCAAGAGAAGGCCATCAAGAGAAGAGAGGCCCTGGAACGGGCCAAGAGAAACGGCAGCATCGTGTCTATGAA
CCTGAAGGACGCCTTCGCCAGATCCATGGAACTGATCGACGTGGCCGTGGAAAAGGCCGGCGAAAGCGCCAATACCAAGGACAGCGCCGACGACAAC
CACCTGTCTCCAAGCAGATGGAAGTGGGCCAGAAAGGCCCTGAGCGAGACAAGCAGCAACAAGTCCTTCGAGAACAAGTACCAAGAGGTGTCCCAGA
AGGACAGCCACGAGCAGCTGAACAACACCTCCAGCTCTAGCCCTCAGCACCTGAGCGCACAGAAACTGGAAATGCTGTACAACGAGATCACCAAGAC
ACAGCCCCACTCTCACCCCAATCCTGACTGCCAAGAAAAGCCCGAAAGACCCAGCGCCTACGAGGAAGAGATCGAAATGGAAGAGGTCGTGTGCCCT
CAAGAGCAGCTGGCTGTGGCTCAGACCGAAGTGATCGTGGACATGAAGTCCACCAGCAGCATCGACAGCTTTACCAGCTGCGCCACCGACTTCACCG
AGACAGAGAGATCTCCACTGCCTCCTCCAAGCGCCAGCCACCTCCAGATGAAGTTCCCTACAGATCTGCCCGGCACCGAGGAACACCAGAGAGCTAG
AGGACCTCCATTTCTGACCCTGAGCAGAGAGAAGGGACCTGCCGCCAGAGATGGCACCCTGGAATATGCCCCTGTGGATATCACCGTGAACCTGGAT
GCCAGCGGCTCTCAGTGTGGACTGCATAGCCCTCTGCAGTCCGACAACGCCACAGACAGCCCTAAGTCTAGCCTGAAGGGCAGCAACCCTCTGAAGT
CTCGGAGCCTGAAAGTGAACTTCAAAGAGAACCGGGGCAGCGCCCCTCAGACACCTCCATCTACAGCTAGACCTCTGCCTGTGACCACCGCCGACTT
CAGCCTGACAACACCACAGCACATCAGCACCATCCTGCTCGAGGAAACCCCTAGCCAAGGCGATAGACCCCTGCTGGGAACCGAAGTGTCTGCCCCT
TGTCAGGGCCCTTCCAAGGGACTGAGCCCTAGATTCCCCAAGCAGAAGCTGTTCCCATTCAGCAGCAGAGAGCGGCGGAGCTTTACCGAGATCGATA
CCGGCGACGACGAGGACTTTCTCGAACTGCCTGGCGCTCGGGAAGAGAAACAGGTGGACAGCAGCCCAAACTGCTTCGCCGATAAGCCAAGCGACGG
CAGAGATCCCCTGAGAGAAGAGGGATCTGTGGGCTCTAGCAGCCCTCAGGATACCGGCCACAACTGCAGACAGGATATCTACCACGCCGTGTCCGAA
GTGAAGAAGGACTCCTCTCAAGAGGGCTGCAAGATGGAAAATCACCTGTTCGCCCCTGAGATCCACAGCAACCCCGGCGATACAGGCTACTGCCCTA
CCAGAGAAACCAGCATG

B

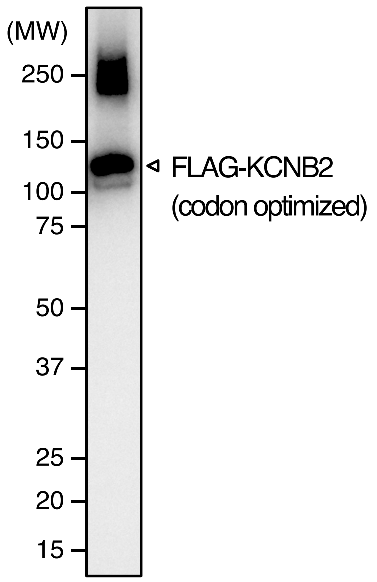

Western blotting: Anti-FLAG mAb-HRP

Figure S1. Codon optimization improved KCNB2 synthesis. (A) Codon-optimized DNA sequence of KCNB2. (B) Western blotting image of codon-optimized KCNB2 protein. FLAG-tagged KCNB2 was detected by horseradish peroxidase conjugated anti-FLAG antibody.
