# Supplementary material for: Evaluation of Cell-Free Synthesized Human Channel Proteins for In Vitro Channel Research
Source: Membranes (Basel). 2022 Dec 30;13(1):48. doi: 10.3390/membranes13010048 (PMC9861611; doi:10.3390/membranes13010048)
Supplement: Supplementary file 1 [file membranes-13-00048-s001.zip › Supplementary files/Table S2.pdf]

Table S2. Summary of PLB assay result.

Symbol	Synonims	Western blotting	PLB assay result
KCNA1	$K_{v}1.1$	Success	Normal current signal
KCNA2	$K_{v}1.2$	Success	Normal current signal
KCNA3	$K_{v}1.3$	Success	Normal current signal
KCNA4	$K_{v}1.4$	Success	Normal current signal
KCNA5	$K_{v}1.5$	Success	Normal current signal
KCNA6	$K_{v}1.6$	Success	No voltage dependent
KCNA7	$K_{v}1.7$	Success	No voltage dependent
KCNA10	$K_{v}1.8$	Success	Temporary opening
KCNB1	$K_{v}2.1$	Success	Normal current signal
KCNB2	$K_{v}2.2$	Not detected	Normal current signal
KCNC1	$K_{v}3.1$	Success	Normal current signal
KCNC2	$K_{v}3.2$	Success	Base line drift
KCNC3	$K_{v}3.3$	Success	Normal current signal
KCNC4	$K_{v}3.4$	Success	Normal current signal
KCND1	$K_{v}4.1$	Success	Normal current signal
KCND2	$K_{v}4.2$	Success	No current signal
KCND3	$K_{v}4.3$	Success	No current signal
KCNH1	$K_{v}10.1$, EAG1	Success	Normal current signal
KCNH2	$K_{v}11.1$, HERG1	Smaller	Normal current signal
KCNH3	$K_{v}12.2$, ELK2	Success	No current signal
KCNH4	$K_{v}12.3$, ELK1	Success	Normal current signal
KCNH6	$K_{v}11.2$, HERG2	Success	Partial opening
KCNH7	$K_{v}11.3$, HERG3	Success	Normal current signal
KCNH8	$K_{v}12.1$, ELK3	Success	Partial opening
KCNMA1	$K_{Ca}1.1$, MaxiK, BK	Larger	Partial opening
KCNJ1	$K_{ir}1.1$	Success	Normal current signal
KCNJ10	$K_{ir}4.1$	Success	No current signal
KCNJ15	$K_{ir}4.2$	Success	Partial opening
KCNJ16	$K_{ir}5.1$	Success	No current signal
KCNK1	$K_{2P}1.1$, TWIK1	Success	No current signal
KCNK2	$K_{2P}2.1$, TREK1	Success	Normal current signal
KCNK5	$K_{2P}5.1$, TASK2	Success	Normal current signal
KCNK6	$K_{2P}6.1$, TWIK2	Success	Normal current signal
KCNK7	$K_{2P}7.1$, KCNK8	Success	Partial opening
KCNK9	$K_{2P}9.1$, TASK3	Success	Partial opening
KCNK10	$K_{2P}10.1$, TREK2	Success	No current signal
KCNK12	$K_{2P}12.1$, THIK2	Success	Temporary opening
KCNK13	$K_{2P}13.1$, THIK1	Success	Temporary opening
KCNK15	$K_{2P}15.1$, TASK5	Success	Normal current signal
KCNK16	$K_{2P}16.1$, TALK1	Success	Temporary opening
KCNK17	$K_{2P}17.1$TASK4	Success	No current signal
KCNK18	$K_{2P}18.1$TRESK1	Success	No current signal
KCNQ1	$K_{v}7.1$	Success	Normal current signal
KCNQ2	$K_{v}7.2$	Success	Normal current signal
KCNQ3	$K_{v}7.3$	Success	Normal current signal
KCNQ5	$K_{v}7.5$	Success	No current signal
KCNT2	$K_{Na}1.2$	Success	No current signal
